# Acute cannabinoids impair association learning via selectively enhancing synaptic transmission in striatonigral neurons

**DOI:** 10.1186/s12915-022-01307-1

**Published:** 2022-05-13

**Authors:** Meilin Wu, Yuanyuan Di, Zhijun Diao, Chuanting Yan, Qiangqiang Cheng, Huan Huang, Yingxun Liu, Chunling Wei, Qiaohua Zheng, Juan Fan, Jing Han, Zhiqiang Liu, Yingfang Tian, Haijun Duan, Wei Ren, Zongpeng Sun

**Affiliations:** 1 MOE Key Laboratory of Modern Teaching Technology, Shaanxi Normal University, Xi’an, 710062 China; 2 School of Psychology, Shaanxi Normal University, Xi’an, 710062 China; 3 College of Life Sciences, Shaanxi Normal University, Xi’an, 710062 China; 4 School of Education, Shaanxi Normal University, Xi’an, 710062 China

**Keywords:** HU210, Reinforcement learning, Learning impairment, D1 MSNs, DREADD, Chemogenetics, Intracranial injection

## Abstract

**Background:**

Cannabinoids and their derivatives attract strong interest due to the tremendous potential of their psychoactive effects for treating psychiatric disorders and symptoms. However, their clinical application is restricted by various side-effects such as impaired coordination, anxiety, and learning and memory disability. Adverse impact on dorsal striatum-dependent learning is an important side-effect of cannabinoids. As one of the most important forms of learning mediated by the dorsal striatum, reinforcement learning is characterized by an initial association learning phase, followed by habit learning. While the effects of cannabinoids on habit learning have been well-studied, little is known about how cannabinoids influence the initial phase of reinforcement learning.

**Results:**

We found that acute activation of cannabinoid receptor type 1 (CB1R) by the synthetic cannabinoid HU210 induced dose-dependent impairment of association learning, which could be alleviated by intra-dorsomedial striatum (DMS) injection of CB1R antagonist. Moreover, acute exposure to HU210 elicited enhanced synaptic transmission in striatonigral “direct” pathway medium spiny neurons (MSNs) but not indirect pathway neurons in DMS. Intriguingly, enhancement of synaptic transmission that is also observed after learning was abolished by HU210, indicating cannabinoid system might disrupt reinforcement learning by confounding synaptic plasticity normally required for learning. Remarkably, the impaired response-reinforcer learning was also induced by selectively enhancing the D1-MSN (MSN that selectively expresses the dopamine receptor type 1) activity by virally expressing excitatory hM3Dq DREADD (designer receptor exclusively activated by a designer drug), which could be rescued by specifically silencing the D1-MSN activity via hM4Di DREADD.

**Conclusion:**

Our findings demonstrate dose-dependent deleterious effects of cannabinoids on association learning by disrupting plasticity change required for learning associated with the striatal direct pathway, which furthers our understanding of the side-effects of cannabinoids and the underlying mechanisms.

**Supplementary Information:**

The online version contains supplementary material available at 10.1186/s12915-022-01307-1.

## Background

Derivatives of cannabinoids or marijuana have potential therapeutical applications for treating multiple psychiatric disorders or symptoms [1, 2]. Despite the recent surge of interest in their potential medical use, the application of these derivatives has been restricted by many side-effects that are related to the dosages used [3] and the physical state of users [4]. For the standardized pharmaceutical application of cannabis derivatives, it is of high importance to investigate the side-effects comprehensively and thoroughly. Extensive studies have demonstrated cannabinoids influence emotional, spatial learning/memory, and working memory through changes in the amygdala, hippocampus, and prefrontal cortex at different levels, notably the related synaptic plasticity changes [5–7]. Since the role of the striatum in operant learning is increasingly emphasized, more and more attention has been paid to the effects of cannabinoids on striatum-dependent learning recently [8, 9].

There are mainly two types of MSNs in the dorsal striatum, the direct pathway MSNs expressing dopamine receptor type 1 (D1R) and the indirect pathway MSNs expressing dopamine receptor type 2 (D2R), which have different projection targets and exert various functions [10]. Different types of neurons in the dorsal striatum which integrate diverse excitatory afferents from the cortex, thalamus, and dense innervation from midbrain dopamine neurons have been suggested to express high levels of CB1Rs [11–13] and exhibit multiple forms of synaptic plasticity mediating learning [8, 14]. It is often suggested that long-term potentiation (LTP) is mainly observed in D1 MSNs which is mediated by N-methyl-d-aspartate receptors (NMDAR), and long-term depression (LTD) mediated by CB1R and metabotropic glutamate receptor usually occur in D2 MSNs [15, 16]. However, various types of endocannabinoid-mediated synaptic plasticity have been observed in both D1 MSNs and D2 MSNs [12, 13, 17]. It has been widely considered that the endocannabinoid (eCB) system unidirectionally depresses neuronal communication on a short or long timescale, while recent reports unveiled that eCB-mediated LTP (eCB-LTP) also plays an important role in learning and memory [18], which is regulated by dopamine via D1R and D2R. The intricate dopamine-endocannabinoid system together with the direct/indirect pathways is widely reported to play a role in reinforcement learning.

Reinforcement learning is one of the most important forms of learning mediated by the striatum [10], which is commonly used as a behavioral intervention and assessment in different psychiatric disorders [19, 20]. It is a process to maximize reward (positive reinforcement learning) and evade aversive stimulus (negative reinforcement learning, NRL), which enables individuals to accumulate the environmental evidence and optimize behavioral strategies. Characterized by an initial response-reinforcer/outcome association phase, followed by a phase of habit (stimulus-response) learning [15, 21], reinforcement learning has been confirmed to be mainly regulated by two subregions of dorsal striatum respectively [21–23]. Acquiring the contingency/association between the response and the reinforcer is dominantly mediated by the dorsomedial striatum [21], while habit learning/expression with the defining feature of insensitivities to reinforcer devaluation and contingency degradation is more supported by the dorsolateral striatum (DLS) [22, 24].

Earlier studies reported that excitatory synaptic changes in the direct pathway mediate the initiation of motion and reward-based learning, while the excitatory synaptic changes of the indirect pathway mediate the inhibition of motion and avoidance-related learning [25]. Recent studies suggested a subtype of D1 MSNs expressing Teashirt family zinc finger 1 (Tshz1) also drive the negative reinforcement [26], and D1 MSNs and D2 MSNs are concomitantly active during reinforcement learning or skill learning but behave differently during performance [27, 28]. Besides, a number of evidences indicated that cannabinoids in DLS play a role in habit learning [29], and eCB-mediated LTD is critical for the shift from goal-directed to habitual responding [9]. Nevertheless, little is known about the role of cannabinoids in DMS in association learning, and whether and how these two sub-types of MSNs are involved in this learning process.

In this study, we firstly investigated the effects of cannabinoids on the initial phase of NRL at different doses and found obvious aversive effects of high-dose HU210 on NRL, which could be imitated by intra-DMS injection of HU210 and alleviated by prior intra-DMS injection of CB1R antagonist AM281. Furthermore, the electrophysiological recordings revealed that administration of HU210 induced enhancement of glutamatergic synaptic transmission in D1 MSNs that occurred after NRL as well, while HU210 disturbed the NRL-related synaptic change, which may underlie the impairment of NRL induced by HU210. Then, we used chemogenetics to specifically inhibit or activate D1 MSNs in DMS and found that inhibiting D1 MSNs could rescue the impairment induced by HU210, while activating D1 MSNs had adverse effects similar to HU210 injection.

## Results

### Acute systemic administration of HU210 induced impairment in association learning

**Fig. 1:**
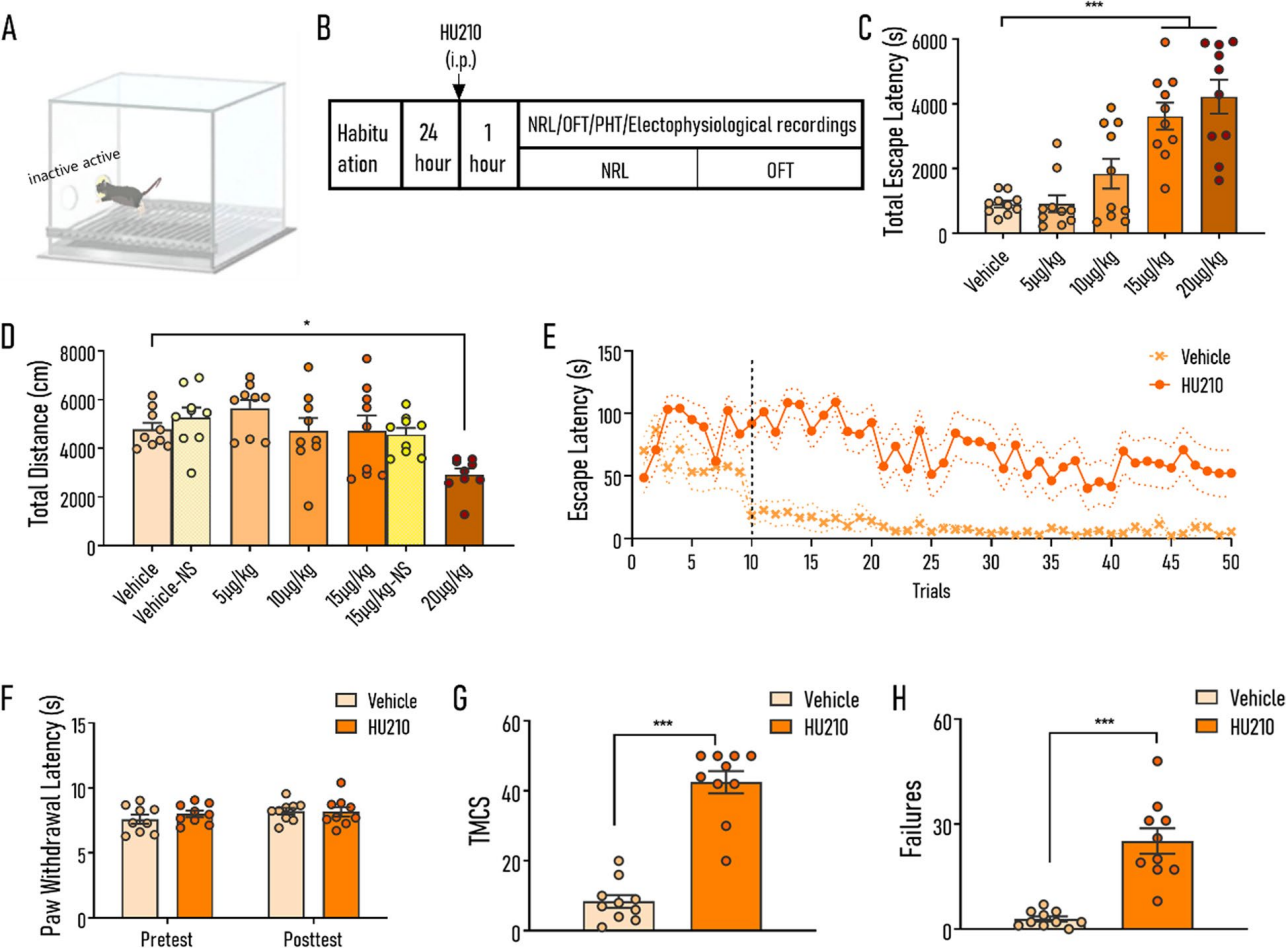
Acute HU210 administration induced dose-dependent impairment in NRL. **A** Schematic representation of the apparatus. **B** The experimental procedure. **C** Acute HU210 administration induced dose-dependent changes in tELS (total escape latencies). **D** Performance of open-field test (OFT) of groups after negative reinforcement learning (NRL), and neither the footshock in learning procedure nor HU210 at a dose of 15 μg/kg had a significant effect on motor activities. **E** Escape latency curves of mice administered with HU210 at a dose of 15 μg/kg (HU210 group) and vehicle. **F** Acute HU210 at a dose of 15 μg/kg had no significant effect on paw algesthesia. **G**, **H** The HU210 group exhibited a significantly increased number of failures and bigger trials to meet the complete success (TMCS) during NRL. Vehicle, mice were administered with vehicle; HU210, mice were administered with HU210 at a dose of 15 μg/kg; -NS, mice did not experience the footshocks of NRL. **C**, **E**, **G**, **H** There were 10 mice in each group. **D**, **F** There were 9 mice in each group. Data shown as mean ± SEM. ANOVA (**C**, **D**, **E**, **F**) and Mann-Whitney test (**G**, **H**): *p < 0.05, ***p < 0.001

Negative reinforcement learning is vital for survival that requires individuals to strengthen the target behavior by removal of negative reinforcers (aversive stimuli). Firstly, the effects of HU210 at four dose gradients (5 μg/kg, 10 μg/kg, 15 μg/kg, and 20 μg/kg) on NRL within one session were assessed (Fig. 1B). Mice injected with HU210 showed hampered NRL in a dose-dependent manner (Fig. 1C). Mice administered with high doses of HU210 (15 μg/kg and 20 μg/kg) displayed significant increases in total escape latencies (tELS, the cumulative latency of the mouse to terminate the footshocks during each session) compared with the vehicle group, while there was no significant difference in tELS between the vehicle group and groups administered with HU210 at doses of 5 μg/kg and 10 μg/kg (treatment *F*_(4,45)_ = 16.07, *p* < 0.001; post hoc test: vs. 5 μg/kg, *p* = 0.989; vs. 10 μg/kg, *p* = 0.322; vs. 15 μg/kg, *p* < 0.001; vs. 20 μg/kg, *p* < 0.001; Fig. 1C). Furthermore, considering the potential influence of HU210 on algesthesia [30] and motor activities [31], we also investigated the effects of HU210 on pain sensitivity and motor activities. The results of the open-field test (OFT) after NRL showed that only the group treated at the dose of 20 μg/kg exhibited a significant decrease in motor activities after the learning procedure (treatment *F*_(4,40)_ = 5.36, *p* < 0.01; post hoc test: vs. 15 μg/kg, *p* = 0.999; vs. 20 μg/kg, *p* < 0.05; Fig. 1D), which implied that the impairment of NRL induced by a moderate dose of HU210 may not be due to its influence on motor activity. To further confirm this conclusion, the effect of HU210 at a dose of 15 μg/kg on traveled distance was examined, and two-way analysis of variance (ANOVA) was used to analyze the main effects of drug treatment and footshock in the learning procedure. The treatment of HU210 at a dose of 15 μg/kg, footshock, and the interaction between them had no significant main effects, indicating that neither footshock experiences nor administration of HU210 affected motor ability (treatment *F*_(1,32)_ = 0.862, *p* = 0.360; stress *F*_(1,32)_ = 0.165, *p* = 0.687; treatment × stress *F*_(1,32)_ = 0.60, *p* = 0.443; Fig. 1D). Moreover, the results of plantar Hargreaves test (PHT) suggested that HU210 at the dose of 15 μg/kg had no significant impact on the algesthesia (treatment *F*_(1,16)_ = 0.316, *p* = 0.582; session *F*_(1,16)_ = 1.980, *p* = 0.179; treatment × session *F*_(1,16)_ = 0.668, *p* = 0.425; Fig. 1F), indicating the impairment of learning may not be due to the sensorimotor deficits. According to the above results, 15 μg/kg was determined to be the optimal dose in the following observation and experiments.

Closer inspection of the learning process revealed that mice treated with HU210 displayed obvious difficulties in acquiring the new instrumental action. As shown in Fig. 1E, escape latencies in each trial (ELS) of the vehicle group fell down to the ground level within 10 trials which indicated a normal association learning, while that of the HU210 group did not show any obvious decrease and maintained at a high level to the end of the whole learning procedure (first 10 trials: treatment *F*_(1,18)_ = 6.958, *p* < 0.05; trial *F*_(9,162)_ = 1.412, *p* = 0.187; treatment × trial *F*_(9,162)_ < 0.01, *p* = 0.208; 11–50 trials: treatment *F*_(1,18)_ = 35.410, *p* < 0.001; trial *F*_(39,702)_ = 2.702, *p* < 0.001; treatment × trial *F*_(39,702)_ = 1.184, *p* = 0.208). Further evidence revealed that the group exposed to HU210 exhibited more escape failures during the whole NRL and required more trials to meet the complete success (TMCS, the trials required to achieve the condition without any failure that is equal to the trial number of the last failure trial before consecutive success) (failures: Mann-Whitney test, *p* < 0.001, Fig. 1F; TMCS: Mann-Whitney test, *p* < 0.001, Fig. 1G). These results suggested systemic HU210 administration could impair the initial phase of reinforcement learning.

### Intra-DMS injection of CB1R antagonist AM281 alleviated the HU210-induced impairment of NRL

**Fig. 2:**
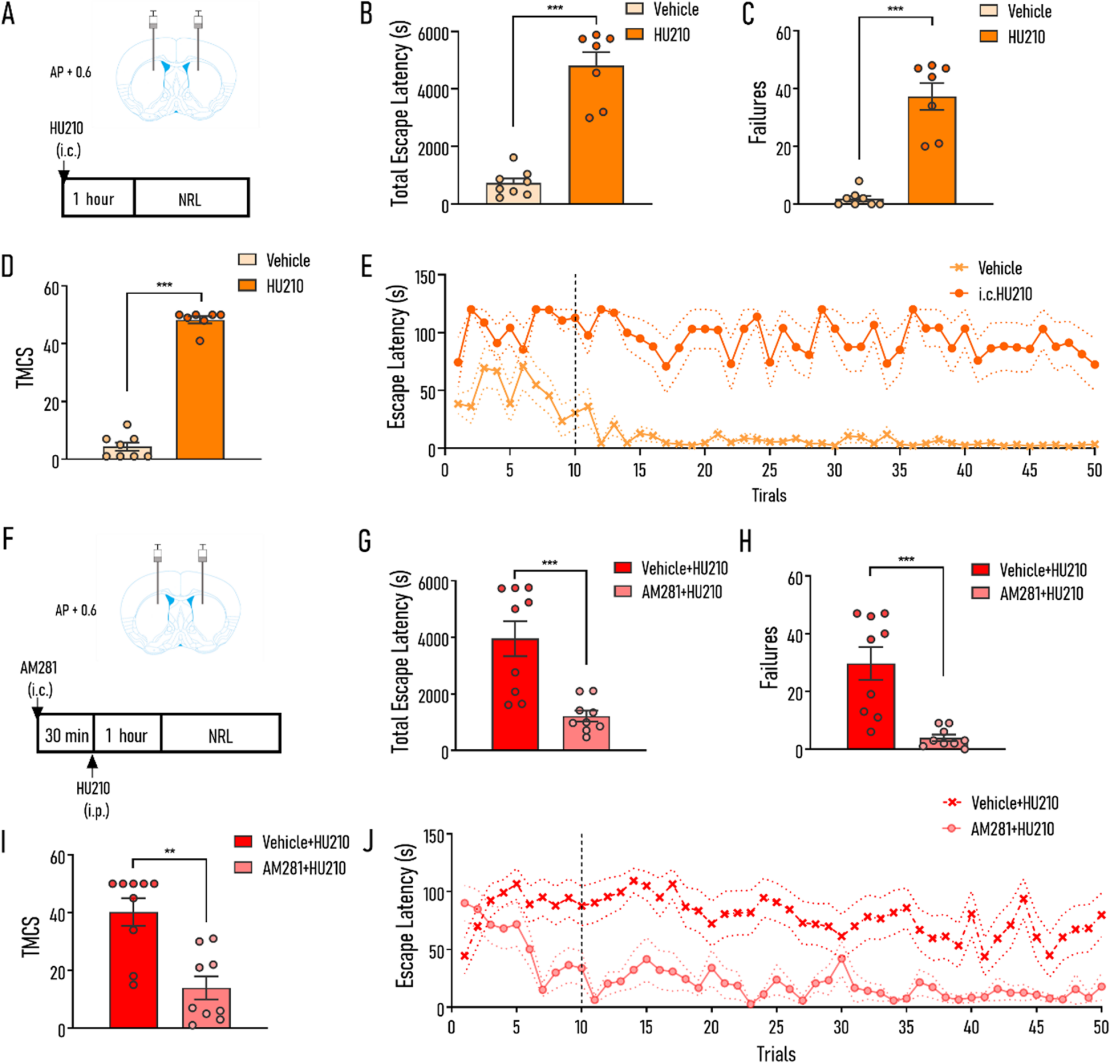
Intra-DMS injection of HU210 and AM281 impaired NPL and alleviated the impairment of NRL, respectively. **A**, **F** A simplified scheme illustrating the injection sites and the experimental paradigms (**A**: intracranial injection of HU210; **F**: combined administration of AM281 and HU210). **B**–**D** Compared with the vehicle group, the HU210 group exhibited significantly increased tELS (**B**), failures (**C**), and TMCS (**D**) during NRL. **E** Escape latency curves of two groups in the intracranial injection of HU210 experiment (vehicle vs. HU210). **G**–**I** Compared with the “Vehicle + HU210” group, the “AM281 + HU210” group exhibited significantly decreased tELS (**G**), failures (**H**), and TMCS (**I**) during NRL. **J** Escape latency curves of two groups (vehicle + HU210 vs. AM281 + HU210). Vehicle, mice were administrated with vehicle (i.c.); HU210, mice were administrated with HU210 (i.c.); Vehicle + HU210, mice were administrated with vehicle (i.c.) and HU210 (i.p.); AM281 + HU210, mice were administrated with AM281 (i.c.) and HU210 (i.p.). HU210, mice were administrated with HU210 (i.c.). Vehicle: *n* = 8; HU210: *n* = 7; Vehicle + HU210: *n* = 9; AM281 + HU210: n = 9. Data shown as mean ± SEM. ANOVA (**E**, **J**), unpaired *t*-test (**B**, **G**), and Mann-Whitney test (**C**, **D**, **H**, **I**): **p < 0.01, ***p < 0.001

In the striatum, CB1Rs are expressed in GABAergic MSNs, astrocytes, and presynaptic nerve terminals of glutamatergic corticostriatal projection [11, 12]. Given the critical role of DMS in reinforcement learning, we subsequently investigated the role of cannabinoid specifically in DMS in learning deficits caused by systemic HU210 injection. To tackle this question, the intra-DMS injection of HU210 was administered (Fig. 2A). We found that intra-DMS administration of HU210 induced a similar impairment of learning to that induced by systemic administration of HU210, including increased tELS (*t*_13_ = 8.655, *p* < 0.001; Fig. 2B), failures (Mann-Whitney test, *p* < 0.001; Fig. 2C), and TMCS (Mann-Whitney test, *p* < 0.001; Fig. 2D). In addition, ELS of the HU210 group maintained at a high level to the end of the whole learning period, which was significantly different from the vehicle group (1–10 trials: treatment *F*_(1,13)_ = 21.990, *p* < 0.001; trial *F*_(9,117)_ = 1.357, *p* = 0.264; treatment × trial *F*_(9,117)_ = 2.487, *p* < 0.05; 11–50 treatment *F*_(1,13)_ = 67.620, *p* < 0.001; trial *F*_(39,507)_ = 1.323, *p* = 0.273; treatment × trial *F*_(39,507)_ = 1.135, *p* = 0.269; Fig. 2E).

To further explore the role of CB1R in DMS in learning disability induced by HU210, specific CB1R antagonist AM281 was injected locally into the DMS with HU210 administered systemically (Fig. 2F). Results showed that combinative administration of AM281 and HU210 significantly alleviated the impairment of learning induced by HU210 injection, including decreased tELS (*t*_16_ = 4.180, *p* < 0.001; Fig. 2G), failures (Mann-Whitney test, *p* < 0.001; Fig. 2H), and TMCS (Mann-Whitney test, *p* < 0.01; Fig. 2I). In addition, ELS of the AM281-treated group decreased significantly after 10 trials while that of the vehicle-treated group maintained at a high level to the end of the whole learning period (1–10 trials: treatment *F*_(1,16)_ = 5.172, *p* < 0.05; trial *F*_(9,144)_ = 1.692, *p* = 0.096; treatment × trial *F*_(9,144)_ = 4.427, *p* < 0.001; 11–50 trials: treatment *F*_(1,16)_ = 17.110, *p* < 0.001; trial *F*_(39,624)_ = 2.271, *p* < 0.001; treatment × trial *F*_(39,624)_ = 1.068, *p* = 0.362; Fig. 2J). These results together with the aforementioned findings of intra-DMS injection of HU210 implied that CB1R in DMS might be implicated in impaired NRL caused by intraperitoneal injection of HU210.

### HU210 intraperitoneal injection altered synaptic transmission in striatonigral MSNs

**Fig. 3:**
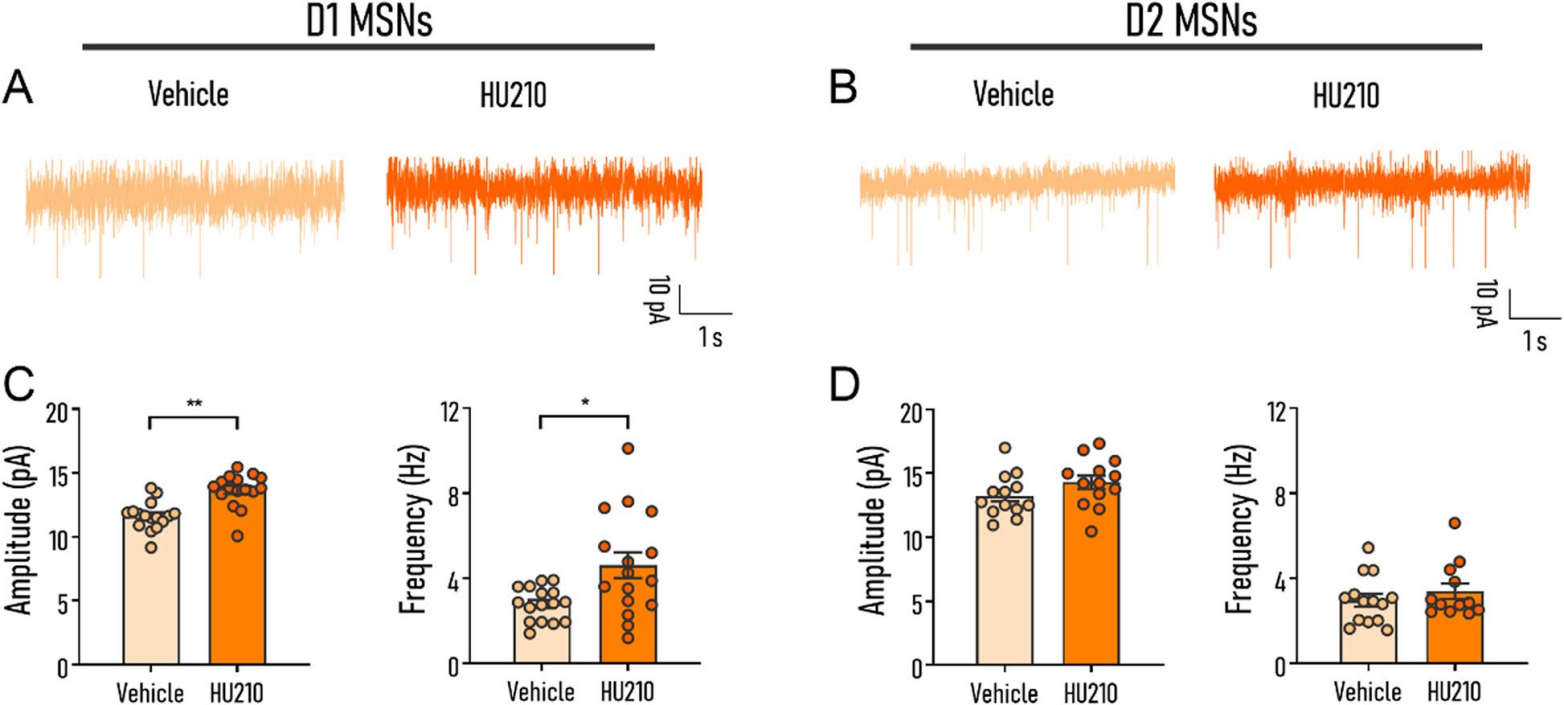
Systemic HU210 administration altered synaptic transmission in D1 MSNs but not in D2 MSNs. **A**, **B** Representative recording traces of mEPSCs of D1 MSNs and D2 MSNs in the vehicle and HU210-treated group, respectively. **C** Summary of mEPSC peak amplitude (pA) (left) and frequency (right) from D1 MSNs of vehicle and HU210 group. **D** Summary of mEPSC peak amplitude (pA) (left) and frequency (right) in D2 MSNs recorded from animals treated with vehicle or HU210. Vehicle, mice were administered with vehicle; HU210, mice were administered with HU210 at a dose of 15 μg/kg. mEPSC frequency: Vehicle D1, *n* = 16, *N* = 5, HU210 D1, *n* = 16, *N* = 5. Vehicle D2, *n* = 14, *N* = 4; HU210 D2, *n* = 12, *N* = 4; mEPSC amplitude: Vehicle D1, *n* = 16, *N* = 5, HU210 D1, *n* = 16, *N* = 5. Vehicle D2, *n* = 13, *N* = 4; HU210 D2, *n* = 13, *N* = 4. *n*: cell number; *N*: animal number. Data shown as mean ± SEM. ANOVA: *p < 0.05, **p < 0.01

Synaptic changes have been widely reported to be associated with the effects of HU210 on the learning process [8]. Thus, we next performed whole-cell voltage clamp recordings to measure miniature excitatory postsynaptic currents (mEPSCs) of D1 MSNs (Fig. 3A) and D2 MSNs (Fig. 3B) that were identified with single-cell RT-PCR (Additional file 1: Fig. S1) to explore the possible cellular and synaptic changes in DMS induced by HU210 injection. HU210 administration induced a significant increase of mEPSCs peak amplitude and frequency (amplitude: Holm-Sidak’s test, *p* < 0.01; frequency: Holm-Sidak’s test, *p* < 0.05; Fig. 3C) in D1 MSNs, while there was no significant difference in D2 MSNs between the HU210-treated group and the vehicle-treated group (amplitude: Holm-Sidak’s test, *p* = 0.206; frequency: Holm-Sidak’s test, *p* = 0.736; Fig. 3D). Besides, there is no significant interaction between the effects of drug administration and neuronal types on amplitude (treatment *F*_(1,54)_ = 15.602, *p* < 0.001; neuron-type *F*_(1,54)_ = 8.009, *p* < 0.01; treatment × neuron-type *F*_(1,54)_ = 1.546, *p* = 0.219) or frequency (treatment *F*_(1,54)_ = 7.374, *p* < 0.01; neuron-type *F*_(1,54)_ = 1.618, *p* = 0.209; treatment × neuron-type *F*_(1,54)_ = 2.801, *p* = 0.100). Therefore, in the following experiments, we focused on the role of D1 MSNs in the impairment of NRL. Intriguingly, it is worth noting that synaptic transmission is also enhanced in D1 MSNs after negative reinforcement learning, similar to the effect of HU210 administration (amplitude: Con vs. NRL *p* < 0.001; frequency: Con vs. NRL *p* < 0.01; Additional file 1: Fig. S2). However, NRL together with HU210 injection compromised the synaptic transmission enhancement (amplitude: Con vs. HU210 + NRL, *p* = 0.083; frequency: Con vs. HU210 + NRL, *p* = 0.405; Additional file 1: Fig. S2), which explains the behavioral results observed in Fig. 1. We further measured the intrinsic properties of D1 MSNs and found that systemic HU210 administration had no significant effect on the rheobase (*t*_10_ = 0.337, *p* = 0.743; Additional file 1: Fig. S3A) and spike numbers (treatment *F*_(1,10)_ = 0.03, *p* = 0.864; current *F*_(12,120)_ = 44.42, *p* < 0.001; treatment × neuron-type *F*_(12,120)_ = 0.422, *p* = 0.952; Additional file 1: Fig. S3B). Meanwhile, by analyzing the calcium imaging data, we found that the activity of D1 MSNs was enhanced significantly after HU210 administration (Mann-Whitney test, *p* < 0.01; Additional file 1: Fig. S4). Taken together, HU210 administration may selectively increase glutamatergic synaptic transmission in D1 MSNs and induce abnormal hyperactivation of D1 MSNs and in turn contribute to the impairment of NRL.

### Activation of D1 MSNs induced impairment of NRL similar to that induced by HU210 administration

**Fig. 4:**
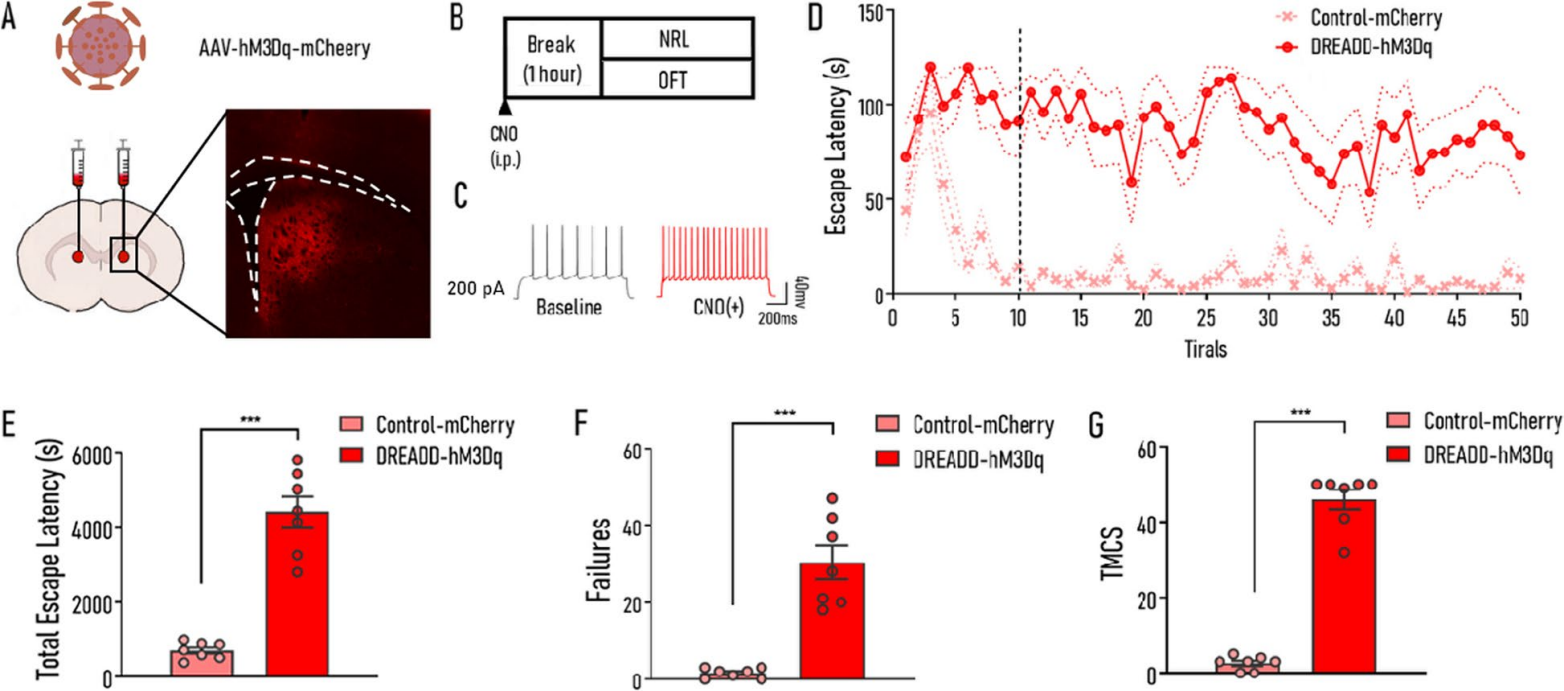
Enhancing D1 MSN activities induced impairment of NRL similar to that induced by HU210 administration. **A** The injection site of rAAV-DIO-hM3Dq-mCherry virus. **B** The experimental procedure. **C** Electrophysiological recordings of representative D1 MSNs infected with hM3Dq virus recorded before and after 10 μmol/L CNO perfusion. **D** Escape latency curves of two groups (Control-mCherry vs. DREADD-hM3Dq)**. E**–**G** Compared with the Control-mCherry group, the DREADD-hM3Dq group exhibited significantly increased tELS (**E**), failures (**F**), and TMCS (**G**) during NRL. Control-mCherry, mice were injected with rAAV-hSyn-DIO-mCherry virus; DREADD-hM3Dq, mice were injected with rAAV-hSyn-DIO-hM3Dq-mCherry virus. Control-mCherry: *n* = 7; DREADD-hM3Dq: *n* = 7. Data shown as mean ± SEM. ANOVA (**D**), unpaired *t*-test (**E**), and Mann-Whitney test (**F, G**): ***p < 0.001

To confirm whether the altered activity of D1 MSNs plays a causative role in the learning deficit induced by HU210 administration, we used the stimulatory designer receptors exclusively activated by designer drugs (DREADD-hM3Dq) (Fig. 4A) to test whether enhancing the activities of D1 MSNs could cause impairment of NRL similar to HU210 (Fig. 4B). To test the functionality of DREADD-hM3Dq in D1 MSNs, we performed whole-cell current-clamp recordings of hM3Dq-mCherry–positive D1 MSNs in acute brain slices and found that D1 MSN exhibited significantly increased spiking response to current stimulation upon bath application of Clozapine-N-oxide (CNO) (Fig. 4C). Then, the NRL performance of mice was evaluated and results showed that an hour after CNO (0.5 mg/kg body weight) i.p. injection, the DREADD-hM3Dq group exhibited obvious impairment of NRL. Similarly, compared with the control group, hM3Dq-expressing mice exhibited significantly increased tELS (*t*_12_ = 8.692, *p* < 0.001; Fig. 4E), failures (Mann-Whitney test, *p* < 0.001; Fig. 4F), and TMCS (Mann-Whitney test, *p* < 0.001; Fig. 4G). In addition, ELS of the DREADD-hM3Dq group maintained at a high level to the end of the whole learning period resembling the HU210-treated group (1–10 trials: virus *F*_(1,12)_ = 65.9, *p* < 0.001; trial *F*_(9,108)_ = 3.844, *p* < 0.01; virus × trial *F*_(9,108)_ = 3.107, *p* < 0.01; 11–50 trial: virus *F*_(1,12)_ = 69.29, *p* < 0.001; trial *F*_(39,468)_ = 1.086, *p* = 0.337; virus × trial *F*_(39,468)_ = 0.729, *p* = 0.729; Fig. 4D). Apart from that, the locomotion of the hM3Dq-expressing mice treated with CNO showed no significant difference in comparison with that of the control group (*t*_10_ = 0.909, *p* = 0.385; Additional file 1: Fig. S5A).

### Inhibition of D1 MSNs after HU210 administration protected against the impairment of NRL

**Fig. 5:**
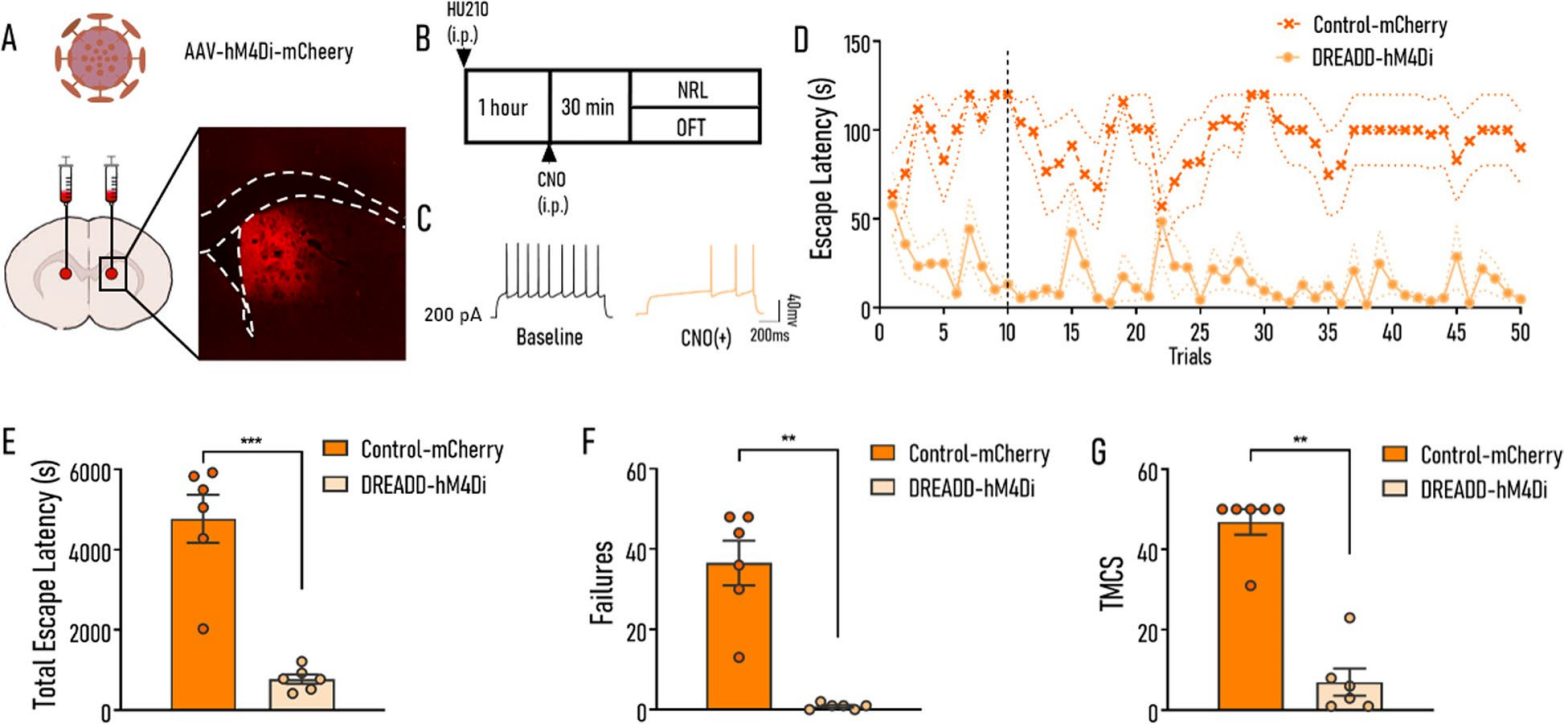
Inhibiting the activity of D1 MSNs after HU210 administration prevents the impairment of NRL. **A** The injection site of AAV-DIO-hM4Di-mCherry virus. **B** The experimental procedure. **C** Electrophysiological recordings of representative D1 MSNs infected with hM4Di virus recorded before and after 10 μmol/L CNO perfusion. **D** Escape latencies curves of two groups (Control-mCherry vs. DREADD-hM4Di). **E**–**G** After CNO and HU210 administration, the DREADD-hM4Di group exhibited significantly decreased tELS (**E**), failures (**F**), and TMCS (**G**) during NRL compared with the Control-mCherry group. Control-mCherry, mice were injected with rAAV-hSyn-DIO-mCherry virus; DREADD-hM4Di, mice were administrated with rAAV-hSyn-DIO-hM4Di-mCherry virus. Control-mCherry: *n* = 6; DREADD-hM4Di: *n* = 6. Data shown as mean ± SEM. ANOVA (**D**), unpaired *t*-test (**E**), and Mann-Whitney test (**F**, **G**): **p < 0.01, ***p < 0.001

To further verify the essential role of D1 MSNs, we used the inhibitory DREADD-hM4Di (Fig. 5A) to investigate whether inhibiting the activity of D1 MSNs could prevent the impairment of NRL induced by HU210 administration (Fig. 5B). The functionality of DREADD-hM4Di in D1 MSNs was examined by whole-cell current-clamp recordings in acute brain slices, and results showed that bath application of CNO decreased responses to current stimulation of hM4Di-mCherry–expressing D1 MSNs significantly (Fig. 5C). On the day of the NRL experiment, mice previously i.p. injected with HU210 at a dose of 15 μg/kg in both the DREADD-hM4Di group and the control group received administration of CNO (1 mg/kg body weight). CNO administration significantly decreased the tELS in DREADD-hM4Di group compared with the control group (*t*_10_ = 6.541, *p* < 0.001; Fig. 5E), together with significantly decreased failures (Mann-Whitney test, *p* < 0.01; Fig. 5F) and TMCS (Mann-Whitney test, *p* < 0.01; Fig. 5G). In addition, there was an improvement in the ESL of the DREADD-hM4Di group within 10 trials, which was not observed in the control group (1–10 trials: virus *F*_(1,10)_ = 61.61, *p* < 0.001; trial *F*_(9,90)_ = 0.7614, *p* = 0.652; virus × trial *F*_(9,90)_ = 2.788, *p* < 0.01; 11-50 trial: virus *F*_(1,10)_ = 37.11, *p* < 0.001; trial *F*_(39,390)_ = 0.697, *p* = 0.916; virus × trial *F*_(39,390)_ = 1.251, *p* = 0.150; Fig. 5D). No significant difference was found in the locomotion test between the hM4Di-expressing mice treated with HU210 and CNO and mice in the control group (*t*_10_ = 2.063, *p* = 0.066; Additional file 1: Fig. S5B). These results further supported that the disturbed synaptic transmission together with the abnormal activation of D1 MSNs may underlie the impairment of NRL induced by HU210.

## Discussion

In the present work, we found that acute HU210 administration affected association learning and motor activities in a dose-dependent manner. The severe impairment of learning mediated by the dysfunctional synaptic enhancement of D1 MSNs could be alleviated by intra-DMS injection of AM281 or inhibition of D1 MSNs, indicating that the normal function of the direct pathway mediated by CB1R is implicated in reinforcement learning.

As previously reported, there was an interaction effect between HU210 administration and stress on motor activities, and deficits in motor activities were only observed in stressed mice administrated with high-dose HU210 [31]. Consistent with this report, our results revealed that mice treated with HU210 at the doses lower than 20 μg/kg displayed no obvious deficits in motor activities in OFT. Moreover, HU210 i.p. injection at a dose of 15 μg/kg did not induce a significant impact on hind paw algesthesia, which is consistent with a prior report using a dosage of 50 μg/kg [32], and previous research indicated that it seemed unlikely that HU210 could affect the perception to the degree that would abolish behavioral performance [33]. Taken together, these results excluded the possibility that the impairment of association learning induced by HU210 injection was due to the locomotor and sensorimotor deficits, which was also supported by the phenomenon that mice treated with HU210 at a dose of 15 μg/kg showed obvious stress responses when they received footshocks as training began (see videos in Additional files 3 and 4). Despite that, the representative performance of mice treated with HU210 showed that they seemed to have deficits in establishing the connection between the reinforcer and the behavior (Additional file 1: Fig. S6), for they were not able to terminate footshocks successfully even after they had experienced a number of successes, which is quite different from that of mice in the vehicle group.

Extensive studies have demonstrated that DMS and DLS mediated the association learning and the following habit learning of reinforcement learning, respectively, whereas a majority of attention had been paid to the role of the cannabinoid system in DLS in habit learning because of the relatively low expression of CB1R in DMS [8, 19]. Considering the critical role of DMS in association learning, it is essential to assess the contribution of CB1R in DMS to reinforcement learning. By injecting the CB1R antagonist AM281 into the DMS of mice with systemic administration of HU210, we found that the impairment of NRL induced by HU210 could be alleviated by intra-DMS injection of AM281, implying that the activation of CB1R in DMS may underlie the association learning impairment. Furthermore, electrophysiological recordings demonstrated that HU210 selectively enhanced synaptic transmission in D1 MSNs in DMS but not D2 MSNs, and calcium imaging indicated the activity of D1 MSNs was also enhanced by HU210, while no obvious change was found in the intrinsic properties of D1 MSNs, suggesting that the enhanced synaptic transmission in D1 MSNs may be a crucial cause for the impairment of NRL. This is further confirmed by the fact that enhancing the activity of D1 MSNs could induce similar impairment of NRL while inhibiting the activity of D1 MSNs after HU210 administration could prevent the impairment of NRL, implying that HU210 may disturb the normal synaptic plasticity and activity of D1 MSNs necessary for learning. Altogether, our current study presented a possible underlying mechanism in which the endocannabinoid system is involved in the regulation of the striatonigral pathway that plays a role in response-reinforcer association establishment.

To the best of our knowledge, this is the first study to provide direct evidence that acute HU210 administration can induce dose-dependent impairment of response-reinforcer association, and explore its possible synaptic mechanisms. It is a little surprising that exogenous cannabinoid induced learning deficits by enhancing synaptic transmission in D1 MSNs in DMS, considering that the CB1Rs are known as inhibitory receptors and the activation of D1 MSNs often facilitates learning. However, activities of dopamine can regulate the emergence of eCB-LTP, which can be elicited in the condition of D1R activation [13]. During the initial phase of reinforcement learning, dopaminergic neurons reacting to the physical property and value of stimulus to encode the reward prediction error increased the release of dopamine to facilitate new action acquisition [34], which in turn may facilitate eCB-LTP. Furthermore, abnormal enhancement of excitatory input or reduction of inhibitory input mediated by CB1R seems candidates to explain the enhanced synaptic transmission in D1 MSNs induced by HU210. As mentioned above, in addition to the expression in MSNs, CB1Rs are expressed in astrocytes in DMS as well, which serves an important role in balancing extracellular glutamate levels through glutamate transporters [35]. A previous study reported that when the function of glutamate transporters was disturbed, enhanced postsynaptic excitation could be recorded at physiological temperature [36]. Thus, a possible mechanism underlying the learning impairment induced by increased excitatory input is that the astroglial CB1R modulation by means of binding of exogenous cannabinoids to CB1Rs on astrocytes in DMS disturbs the glutamate uptake by astrocytes and gives rise to the increased extracellular glutamate levels [7], and then abnormal synaptic enhancement (induced by HU210) of D1 MSNs resulted in altered sensitivity to excitatory presynaptic inputs (associated with learning) and in turn gave rise to the deficits in associating the nosepoking and the termination of footshock (a reward). Accordingly, enhancement of synaptic transmission in D1 MSNs inducing impairment of learning may share the mechanism with postexcitatory depression, which refers to the phenomenon that the high frequency of excitatory input can suppress the conduction of action potentials along axons, leading to neural inexcitability and conduction failures [37]. For example, experience or electrical stimulation protocol can induce LTP in the hippocampus; however, if the glutamatergic transmission is enhanced to a potentiated level aforehand, it would induce depotentiation and reverse the established potentiation to the previous baseline transmission level [38, 39]. That means the enhanced synaptic transmission of D1 MSNs elicited by exogenous cannabinoid may conflict with the physiological enhancement of synaptic transmission required for NRL, which eventually resulted in decreased synaptic transmission of D1 MSNs. This speculation was supported by our results that both the exogenous cannabinoids and the reinforcement learning could elicit the enhancement of mEPSC in D1 MSNs, while the combination of these two factors induced significant depression of synaptic transmission of D1 MSNs (amplitude: Con vs. NRL, *p* < 0.001; HU210 + NRL vs. NRL, *p* < 0.01; frequency: Con vs. NRL, *p* < 0.01; HU210 + NRL vs. NRL, *p* < 0.05; Additional file 1: Fig. S2). Meanwhile, we found that intra-DMS injection of AM281 could only alleviate but not completely abolish the impairment of learning induced by HU210 injection, indicating there may exist other targets of HU210 which also affected the D1 MSNs and in turn induced learning deficits. The mechanisms remain to be elucidated in further studies.

Apart from that, some previous studies reported that activating D1 MSNs prompted locomotion [40], and CNO administration at a dose of 0.7 mg/kg showed a significant inhibitory effect on locomotion [41]. According to our results, activating D1 MSNs of hM3Dq-expressing mice by CNO at a dose of 0.5 mg/kg showed no obvious effect on locomotion [42]. Nevertheless, there was only a tendency for inhibition of D1 MSNs of hM4Di-expressing mice by CNO to exhibit adverse effect on locomotion, which implies that locomotion may not be the crucial factor of learning deficits induced by HU210. Even if the locomotion were to some extent affected in the case of chemogenetic inactivation, these mice were capable to learn the task. Besides, the selective effects of cannabinoids on D1 MSNs should be considered as well, which may participate in astroglial CB1R modulation via NMDARs that play a role in D1-MSN-mediated LTP [15]. These issues should be clarified in future works.

## Conclusions

In summary, our data demonstrate the dose-dependent deleterious effects of cannabinoids on the initial phase of reinforcement learning by disturbing the enhancement of synaptic transmission normally required for reinforcer-response association. These findings have important implications for our understanding of the side-effects of cannabinoids and the underlying mechanisms and provide insights into the interaction of cannabinoid and dopamine systems in regulating basal ganglia-related learning.

## Methods

### Subjects

Male C57BL/6J (7–8 weeks old, from the Model Animal Research Center of Nanjing University, China) and Drd1-Cre mice (5–6 weeks old for virus injection experiments and 8–9 weeks old for behavioral and electrophysiological experiments, kind gift from the laboratory of Xu Fuqiang) were used in the experiments. All experiments were carried out in accordance with the requirements of the Chinese Council on Animal Care and approved by the Animal Care Committee of Shaanxi Normal University. Mice were group housed at 22 ± 2 °C and 55 ± 5% relative humidity under a 12/12 light/dark cycle with food and water ad libitum. All behavioral experiments were conducted during the light part of the cycle. Before the behavioral experiments, mice were gently handled for at least 5 days to minimize manipulation-related stress.

### Negative reinforcement learning to escape footshocks

Negative reinforcement learning experiments were conducted in operant chambers (30 × 24 × 30, L × W × H in cm; MED-Associates, St. Albans, VT), which were equipped with a ventilation fan, a light (4lx), a transparent door, and two nosepokers located 2 cm above the metal grid floor (Fig. 1A). Animals were trained to cease the footshock delivered through the metal grid floor by poking their noses into one of the two nosepokers that was randomly designated as “active” to cease the footshocks. During the shock period, the active nosepoker was illuminated by a light-emitting diode (LED, 20lx) while the inactive nosepoke hole was always not illuminated which has no programmed effects associated with its nosepoking.

Twenty-four hours before the experiment, mice were allowed to acclimate the experimental environment freely for 100 min with no shock. On the day of the experiment, animals were put into the chambers an hour after the HU210 or vehicle injection (the general behavioral experiment procedure is shown in Fig. 1A). The learning procedure consisted of 50 trials. During each trial, continuous mild electric footshocks (0.15 mA) were delivered at the beginning of the experimental program and terminated once the active nosepokers were triggered, or at the time of the maximum shock duration (120 s) when animals failed to terminate the shocks. When the shock was terminated, the LED lights would be turned off, with a 1.5-s tone (2.9 kHz, 65 dB) appearing. Between trials, animals were allowed to rest for a pseudorandom period ranging from 30 to 60 s [43].

### Open-field test

To assess the possible effects of HU210 injection on motor ability, we evaluated locomotor activity an hour after HU210 injection at a dose of 15 μg/kg, or 30 min after NRL in OFT. For DREADD experiments, OFT was performed after CNO injection to evaluate the effects of chemogenetic manipulation on locomotion. Animals were placed individually in the center of a square box (25 × 25 × 50, L × W × H in cm). The moving trajectories were recorded for 10 min with a video camera positioned above the box and analyzed with the EthoVision XT software. Locomotor activity was assessed by the total distance traveled.

### Plantar Hargreaves test

To assess the possible effects of HU210 injection on algesthesia, pain sensitivity was measured by heating the hind paws with the Hargreaves radiant heat apparatus (IITC Life Sciences) [44]. Mice were placed in a bottomless clear plastic box on a glass floor and allowed to acclimate for at least 40 min before the test. An hour after drug or vehicle administration, the radiant heat source was positioned under the glass floor and applied to the plantar surface of the hind paw with an 25% active intensity. The duration from the onset of heating to the first occurrence of one of the following behaviors was recorded as paw withdrawal latency (seconds): jumping, licking the heated paw, or lifting the heated paw, and the maximum heating duration was set as 20 s to prevent tissue damage. Each paw (with 15-min intervals) was tested 3 times to get the averaged paw withdrawal latency. After every experiment, the apparatus was thoroughly cleaned with 70% ethanol.

### Drugs and acute intraperitoneal injection

HU210 and AM281 were purchased from Sigma (USA) and dissolved in vehicle of 2:1:37 of dimethyl sulfoxide (DMSO): Tween-80: 0.9% saline. All drugs and vehicle were dispensed on the day of experiments. HU210 was acutely administrated an hour before the behavioral or electrophysiological experiments by intraperitoneal injection.

Tetrodotoxin (TTX) and picrotoxin (PTX) were purchased from Sigma (USA), which were made into concentrated stock solutions and diluted in artificial cerebrospinal fluid (ACSF) to the final concentration on the day of testing, and continuously poured into the recording chamber. CNO (purchased from BrainVTA, China) was pre-dissolved in DMSO and then diluted with 0.9% saline to a final concentration of 0.5% just before the experiments. For drug stocks prepared with DMSO, the final DMSO concentration was less than 0.1%.

### Intracranial implantation and microinjections

C57BL/6J mice were anaesthetized with 0.8–1.5% isoflurane and fixed in a stereotaxic apparatus (SR-5; Narishige, Tokyo, Japan). To prevent eye injury, the ophthalmic ointment was applied after anesthesia. Guide cannulas (23 gauge with stylets) made of stainless steel tubing were implanted bilaterally into the dorsomedial striatum (AP: +0.6 mm; ML: ±1.5 mm; DV: −2.8 mm). After surgery, mice were allowed to recover for 7 days before the behavioral experiments. Intracranial infusion was administered using an injection needle (30 gauge) inserted through the guide cannula with the injection needle connected to 0.5-μL syringes with polyethylene tubes and controlled by an automated microinjection pump (World Precision Instruments, Sarasota, FL, USA). AM281 solution (0.3 mg/ml) of a total volume of 1.0 μL/mouse (0.5 μL/side) was injected half an hour before the HU210 intraperitoneal injection at a rate of 0.1 μL/min, or HU210 solution (0.2 mg/ml) with the same volume of AM281 was injected an hour before the learning experiment. After injection, the needles were left in place for an extra 3 min for drug diffusion. At the end of the experiment, mice were sacrificed under an overdose of urethane, and the brains were sliced to verify the cannula placements. The data were abandoned if the cannula tip was away from the target by > 0.5 mm.

### Surgery and virus injections

Drd1-Cre mice were anesthetized with 0.8–1.5% isoflurane and received injections in a stereotaxic apparatus. To prevent eye injury, the ophthalmic ointment was applied after anesthesia. For the calcium imaging experiment, rAAV-hSyn-DIO-GCaMp6m-WPRE-pA (BrainVTA; approximate titer 5×10^12^ vg/ml) virus solution (200 nL) was injected unilaterally into DMS (AP: +0.6 mm, ML: −1.5 mm, DV: −2.8 mm) using an injection micropipette attached to a nanoinjector at speed of 30 nL per minute. Then, the micropipette was slowly withdrawn after the nanoinjector retained for 10 min 0.03 mm above the injection sites for 10 min for virus diffusion, and an optical fiber (200 μm core diameter, 0.37 numerical aperture (NA); Shanghai Fiblaser) was implanted and secured to the skull with dental cement.

For the DREADD experiments, chemogenetic activation and inactivation were achieved using hM3Dq DREADD (hM3Dq: the excitatory modified Gq-coupled human M3 muscarinic receptor expressed in AAV viral vectors, which can be exclusively activated by CNO) and hM4Di DREADD (the inhibitory modified Gi/o-coupled human M4 muscarinic receptor expressed in AAV viral vectors, which can be exclusively activated by CNO), respectively. In the case of DREADD activation, rAAV-hSyn-DIO-hM3Dq-mCherry virus (mCherry: mCherry fluorescence protein) (BrainVTA; approximate titer 2×10^12^ vg/ml) was bilaterally injected at the following coordinates for each mouse: AP: +1.18 mm, ML: ±1.2 mm, DV: −3.0 mm; AP: +0.6 mm, ML: ±1.5 mm, DV: −2.8 mm. For DREADD activation and control, rAAV-hSyn-DIO-hM4Di-mCherry and rAAV-hSyn-DIO-mCherry viruses are injected, respectively. A total volume of 120 nl was injected at each desired depth at the speed of 30 nl per minute. After every injection, the nanoinjector was retained 0.03 mm above the injection sites for 10 min, and then, incisions were sewed up with sterilized surgical suture after the micropipette was withdrawn.

After surgery, animals were allowed to recover in their home cages for 3 weeks before behavioral experiments. At the end of the experiments, mice were sacrificed with a urethane overdose, and the brains were sliced to verify the virus infection regions.

### Fiber photometry calcium imaging and data analysis

Fiber photometry was used to record the population activity of neurons expressing the genetically encoded calcium indicator in real time. The light between the commutator and the implanted optical fiber was guided by an optical fiber (200-μm core diameter, 0.37 NA; 2 m long), and the laser intensity at the tip of the optical fiber was measured and adjusted to 10–20 μW to minimize photobleaching. The signals were collected by the multi-channel fiber photometry recording system (Thinkertech) and digitalized and recorded at 50 Hz by ThorCam-DAQ. Recordings were performed in open field as described above and last for 5 min. Before recording, mice were allowed to move freely for 5 min to acclimate.

After recording, data were processed using custom-written MATLAB software. The fluorescence change (d*F*/*F*) was estimated using (*F*(*t*) − *F*0)/*F*0, where (*F*(*t*) − *F*0) was calculated by subtracting the median fluorescence value of the whole session (*F*0) from the fluorescence value at each time point (*F*(*t*)) [45]. Only peaks whose amplitudes exceeded the median average deviation by 2.91 deviations were included in the peak event analysis [46].

### Electrophysiological recordings

An hour after the HU210 or vehicle injection, mice were anaesthetized with isoflurane and decapitated. The forebrain was separated from the cerebellum by a coronal cut, divided into two parts by a sagittal cut from the longitudinal fissure, and immediately glued to a cutting stage immersed in oxygenated (95% O_2_ and 5% CO_2_) ACSF at physiological temperature (~ 34°C) containing (in mM): 125 NaCl, 2.5 KCl, 25 glucose, 25 NaHCO_3_, 1.25 NaH_2_PO_4_, 2 CaCl_2_, and 1 MgCl_2_ (pH 7.2-7.4). Sagittal slices (300 μm) through the striatum were quickly cut with a vibratome (VT 1200S, Leica, Germany) at a speed of 0.06 mm/s. Then, slices were incubated in the oxygenated ACSF for 30 min at 32 ~ 34°C to recover for 1 h. After recovering, a slice was transferred to a recording chamber and perfused with continuously oxygenated ASCF at 31 ± 1 °C. The recording pipettes had a resistance of 3–5 MΩ when filled with the RNase-free solution below (in mM): 140 Cs-methane sulfonate, 2 MgCl_2_, 0.2 EGTA, 10 HEPES, 4 Mg-ATP, 0.3 (Na_2_) GTP, 2 QX-314, 10 Na_2_-phosphocreatine (pH 7.2–7.4 with CsOH). MSNs in the dorsal striatum were visualized using an upright microscope (DM LFSA, Leica, Germany). Neurons were preliminarily selected and identified by their morphological features (flat appearance, medium size, large initial axon segment), and whole-cell recordings were carried out with a Multiclamp 700B amplifier (Molecular Devices, Sunnyvale, CA, USA). mEPSCs were recorded at a holding potential of −80 mV in the ACSF superfusate with 100 μM PTX and 1 μM TTX. Continuous recordings of mEPSCs for least 5 min were filtered at 2 kHz and digitized at 10 kHz using a Multiclamp 700B amplifier and a Digidata 1550 (Molecular Devices, USA). Recorded mEPSCs were analyzed using Clampfit (Molecular Devices, Sunnyvale, CA, USA) and detected based on a template-matching algorithm (threshold: amplitude > 4 pA; baseline: − 80 mV). The intrinsic properties were examined by current-clamp recording. Currents were set in steps of 25 pA, ranging from −200 to 300 pA, 1000ms duration, and neurons were recovered for 5 min before recording.

For function test of expressed hM3Dq and hM4Di protein, current-clamp recording was applied to measure evoked action potentials in CNO activation or inhibition experiment. D1 MSNs expressing hM3Dq or hM4Di were visually identified by mCherry. Current-clamp recording protocol was the same as aforementioned. Neurons were recovered before the brain slices were perfused with ACSF containing 10 μM CNO, and the same current-clamp procedure was performed 10 min after CNO perfusion.

### Single-cell RT-PCR

To identify the type of recorded MSNs, single-cell RT-PCR was performed. After electrophysiological recording, a small negative pressure was applied to the patch pipette to attach the neuron. The pipette was gently withdrawn to pull the neuron off the slice. Then, the electrode with the attached neuron was put into a microcentrifuge tube containing 3 μL ddH_2_O and 0.5 μL of 40 U/μL RNasin (Promega, USA); subsequently, reverse transcription-polymerase chain reaction (RT-PCR) assay was performed to acquire its cDNA. The acquired cDNA fragments were amplified twice by nest PCR and subjected to 2% AGAR gel electrophoresis. Single-cell reverse transcription and PCR amplification were based on a patented method (see the supplementary methods and tables) [42]. All PCR reagents were from Takara (Japan) and the representative image of the agarose gel electrophoresis is shown in Additional file 1: Fig. S1.

Single-strand cDNA was synthesized in PCR tubes containing 2 μL mixed dNTPs (2.5 mmol/L each), 0.5 μL oligo (dT) primer (50 μmol/L), and 0.5 μL random primer (100 μmol/L) (Takara, Japan). The mixture was heated to 65 °C for 5 min and then chilled on ice for 1 min. After chilling, 2.5 μL 5 × RT Buffer and 0.75 μL Maxima Reverse Transcriptase (200 U/μL; Thermo Scientific, USA) were added and the mixture was held at the temperature of 25 °C for 10 min, 50 °C for 30 min, and 85 °C for 5 min, and finally kept at 4 °C. A multiplex single-cell nested-PCR was carried out for identification of dopamine receptor type of MSNs (Drd1 for D1, Drd2 for D2, GAD67 for GABA). Primers and amplicons are shown in Additional file 2 (Table S1), and the PCR reaction conditions are shown in Additional file 2 (Table S2). The second-round PCR products were identified by 2% agarose gel electrophoresis.

### Histology

After calcium imaging and DREADD experiments, mice were deeply anesthetized with sodium pentobarbital (50 mg in a volume of 0.1 ml) and transcardially perfused with 5–10 ml 9% saline, followed by 10–15 ml chilled 4% paraformaldehyde. Brains were removed and post-fixed overnight at 4°C for 24 h, followed by dehydration in a solution of 30% sucrose in 0.1 M PBS for at least 2 days. Forty-micrometer coronal slices were prepared on a freezing microtome (CM1950, Leica Microsciences, Germany), and fluorescence images were taken with a laser scanning microscope (Leica, TCS SP5, Germany).

### Statistical analysis

ANOVA for trial and current with repeated measures, unpaired *t*-test, and Mann-Whitney test were used to determine the difference between groups. Post hoc testing was conducted using Holm-Sidak’s test for multiple comparisons. Differences with *p* < 0.05 were considered statistically significant. All statistical analyses were conducted using SPSS version 20 (IBM Corp., Armonk, NY).

### Availability of data and materials

All data generated or analyzed during this study are included in this published article and its additional files.

## Supplementary Information

**Additional file 1: Figure S1.** Representative agarose gel electrophoresis image of single-cell PCR. **Figure S2.** Summarized data of mEPSC peak amplitude and frequency from D1 MSNs with different treatments. **Figure S3.** Systemic HU210 administration had no effect on intrinsic properties of D1 MSNs. **Figure S4.** Systemic HU210 administration enhanced the calcium signal of D1 MSNs in DMS. **Figure S5.** The locomotion performance of mice in DREADD experiments. **Figure S6.** Two exemplary learning sessions of mice treated with HU210.

**Additional file 2: Table S1.** Oligonucleotide primers and amplicons used in single-cell PCR. **Table S2.** PCR reaction conditions.

**Additional file 3.** A mouse treated with vehicle showed obvious stress responses when receiving footshocks as training began.

**Additional file 4.** A mouse treated with HU210 at a dose of 15 μg/kg showed obvious stress responses when receiving footshocks as training began.

**Additional file 5.** Individual data values and statistics for Figs. 1, 2, 3, 4 and 5 and Figure S2-S5.

## Data Availability

All data generated or analyzed during this study are included in this published article and supplementary information files. Individual data values and statistics are provided in Additional file 5.

## Abbreviations

CB1R: Cannabinoid receptor type 1 (CB1R)
DMS: Dorsomedial striatum
MSNs: Medium spiny neurons
D1-MSN: MSN that selectively expresses the dopamine receptor type 1
DREADD: Designer receptor exclusively activated by a designer drug
D1R: Dopamine receptor type 1
D2R: Dopamine receptor type 2
LTP: Long-term potentiation
NMDAR: N-Methyl-d-aspartate receptors
LTD: Long-term depression
mGluR: Metabotropic glutamate receptor
eCB: Endocannabinoid
eCB-LTP: eCB-mediated LTP
NRL: Negative reinforcement learning
DLS: Dorsolateral striatum
Tshz1: Teashirt family zinc finger 1
tELS: Total escape latencies
OFT: Open-field test
ANOVA: Analysis of variance
PHT: Plantar Hargreaves test
ELS: Escape latencies in each trial
TMCS: Trials to meet the complete success
mEPSCs: Miniature excitatory postsynaptic currents
CNO: Clozapine-N-oxide
DMSO: Dimethyl sulfoxide
TTX: Tetrodotoxin
PTX: Picrotoxin
ACSF: Artificial cerebrospinal fluid
