# Supplementary material for: Acute cannabinoids impair association learning via selectively enhancing synaptic transmission in striatonigral neurons
Source: BMC Biol. 2022 May 13;20:108. doi: 10.1186/s12915-022-01307-1 (PMC9102575; doi:10.1186/s12915-022-01307-1)
Supplement: Supplementary file 1 — Additional file 1: Figure S1. Representative agarose gel electrophoresis image of single-cell PCR. Figure S2. Summarized data of mEPSC peak amplitude and frequency from D1 MSNs with different treatments. Figure S3. Systemic HU210 administration had no effect on intrinsic properties of D1 MSNs. Figure S4. Systemic HU210 administration enhanced the calcium signal of D1 MSNs in DMS. Figure S5. The locomotion performance of mice in DREADD experiments. Figure S6. Two exemplary learning sessions of mice treated with HU210. [file 12915_2022_1307_MOESM1_ESM.docx]

**Supplementary Information**

Acute cannabinoids impair association learning via selectively enhancing synaptic transmission in striatonigral neurons

Meilin Wu, Yuanyuan Di, Zhijun Diao, Chuanting Yan, Qiangqiang Cheng, Huan Huang, Yingxun Liu, Chunling Wei, Qiaohua Zheng, Juan Fan, Jing Han, Zhiqiang Liu, Yingfang Tian, Haijun Duan, Wei Ren, Zongpeng Sun

**Supplemental Figures**

**
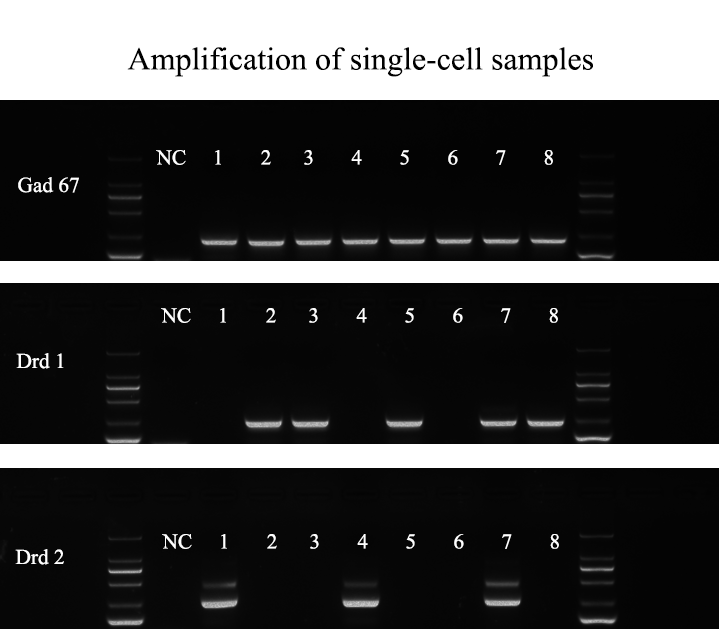
Fig. S1. Representative agarose gel electrophoresis image of single-cell PCR with nested primers applied to single GABAergic (glutamic acid decarboxylase 67, Gad67) MSNs with D1R (Drd1) and D2R (Drd2).** NC: Negative control. Double-positive cells expressing both receptors in single-cell PCR were not counted.

**
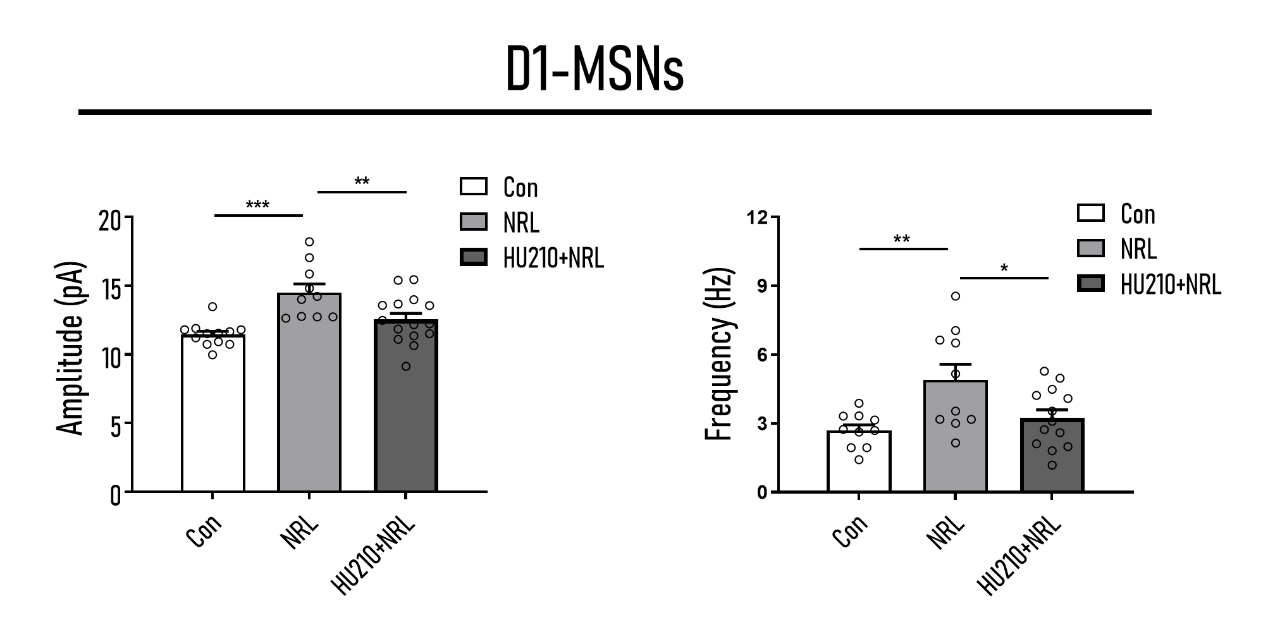
Fig. S2. Summarized data of mEPSC peak amplitude (pA) (left) and frequency (right) from D1 MSNs of control, NRL and HU210+NRL group.** Normal negative reinforcement learning enhanced synaptic transmission in D1 MSNs in general, and after NRL, mice treated with HU210 exhibited significantly decreased synaptic transmission of D1 MSNs (Amplitude *F*_(2,34)_ = 10.30, *p* ˂ 0.001; post hoc test: Con vs. NRL, *p* ˂ 0.001; HU210 + NRL vs. NRL, *p* ˂ 0.01; Con vs. HU210 + NRL, *p* = 0.083; Frequency *F*_(2,30)_ = 5.846, *p* ˂ 0.01; post hoc test: Con vs. NRL, *p* ˂ 0.01; HU210 + NRL vs. NRL, *p* ˂ 0.05; Con vs. HU210 + NRL, *p* = 0.405). Control: Naïve mice without any treatment; NRL: Mice completed the negative reinforcement learning; HU210 + NRL: Mice previously administrated with HU210 and completed the negative reinforcement learning. mEPSC amplitude: Con n = 12, N = 4; NRL, n = 10, N = 4; HU210 + NRL, n = 15, N = 5; mEPSC frequency: Con n = 10, N = 4; NRL, n = 10, N = 4; HU210 + NRL, n = 13, N = 5. n: cell number; N: animal number. Data shown as mean ± SEM. ANOVA: **p* ˂ 0.05, ***p* ˂ 0.01, ****p* ˂ 0.001.

**
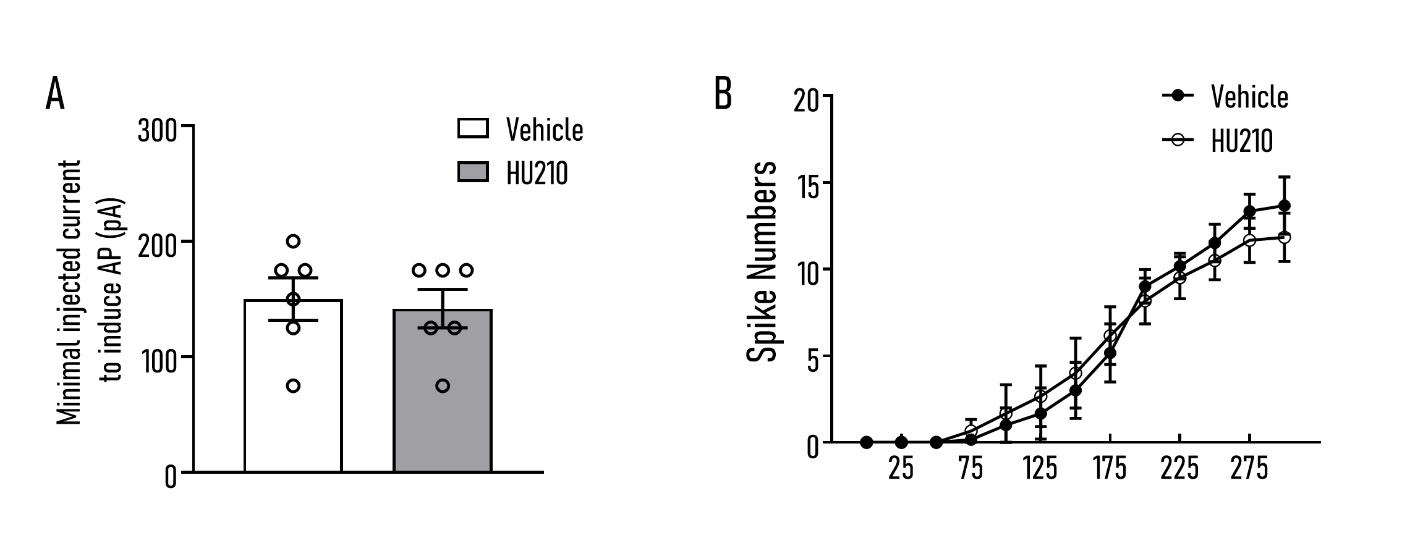
**

**Fig. S3. Systemic HU210 administration had no effect on intrinsic properties of D1 MSNs. A** There was no significant difference in rheobase of minimal injected current to induce action potentials (Aps) between two groups. **B** There was no significant difference in numbers of Aps induced at different current steps between two groups. Vehicle: Mice were administered with vehicle; HU210: Mice were administered with HU210 at a dose of 15 μg/kg. Vehicle, n = 6, N = 3; HU210, n = 6, N = 3; n: cell number; N: animal number. Data shown as mean ± SEM. Unpaired t-test (Rheobase) and two-way ANOVA (Spike Numbers) were used.

**
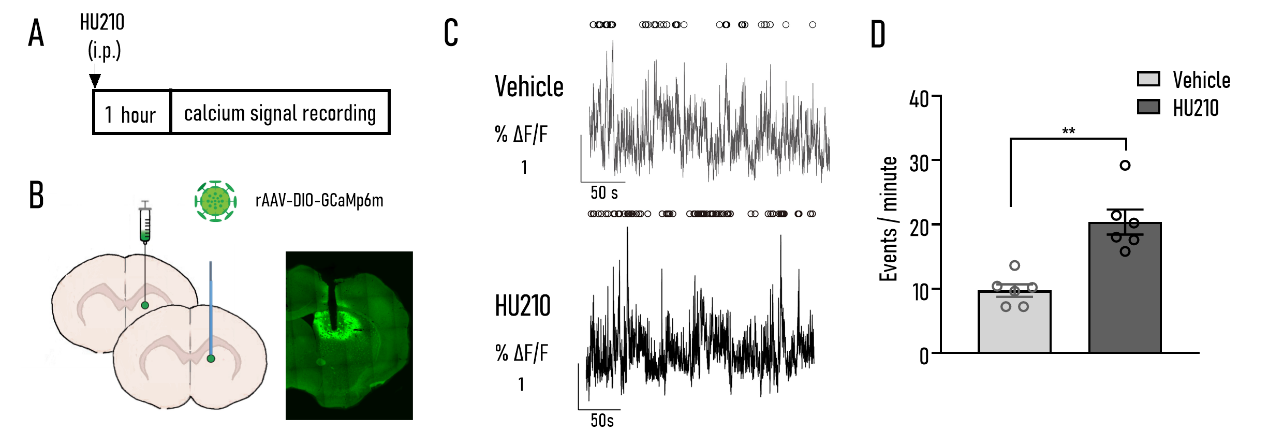
Fig. S4.** **Systemic HU210 administration enhanced the calcium signal of D1 MSNs in DMS. A** The experimental procedure. **B** Injection site of rAAV-hSyn-DIO-GCaMp6m-WPRE-pA virus and location of optical fiber. **C** Representative Ca2+ traces of D1 MSNs of mice injected with vehicle (upper) and HU210 (lower) when freely moving in the open field. Peaks of Calcium transients are illustrated by circles. **D** Peak analysis of calcium imaging traces revealed that systemic HU210 administration significantly increases events of D1 MSNs in DMS. Vehicle: Mice were administered with vehicle; HU210: Mice were administered with HU210 at a dose of 15 μg/kg. Vehicle: n = 6; HU210, n = 6. Data shown as mean ± SEM. Mann-Whitney test: **p* ˂ 0.01.

**
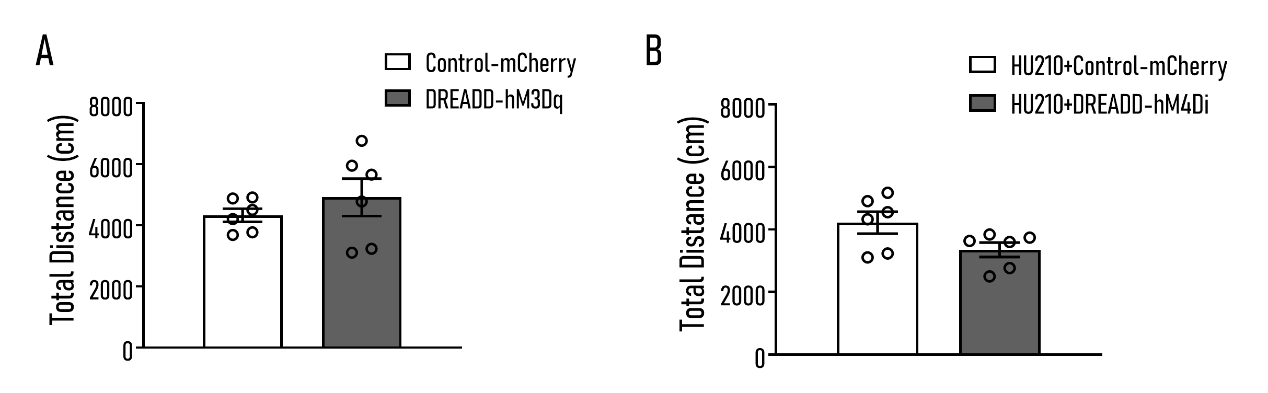
F****ig. S5.** **The locomotion performance of mice in DREADD experiments. A** There was no significant difference in total distance in OFT between mice expressing hM3Dq and mice in control group after CNO injection. **B** There was no significant difference in total distance in OFT between mice expressing hM3Dq and mice in control group after CNO injection. Control-mCherry: Mice were injected with control virus (rAAV-hSyn-DIO-mCherry) and CNO (i.p.); DREADD-hM3Dq: Mice were injected with rAAV-hSyn-DIO-hM3Dq-mCherry virus and CNO (i.p.). HU210+Control-mCherry: Mice with control virus were injected with CNO after HU210 intraperitoneal injection; HU210+DREADD-hM4Di: Mice expressing hM4Di were injected with CNO after HU210 intraperitoneal injection. Control-mCherry: n = 6; DREADD-hM3Dq: n = 6; HU210+Control-mCherry: n = 6; HU210+DREADD-hM4Di: n = 6. Data shown as mean ± SEM. Unpaired t-test was used.

**
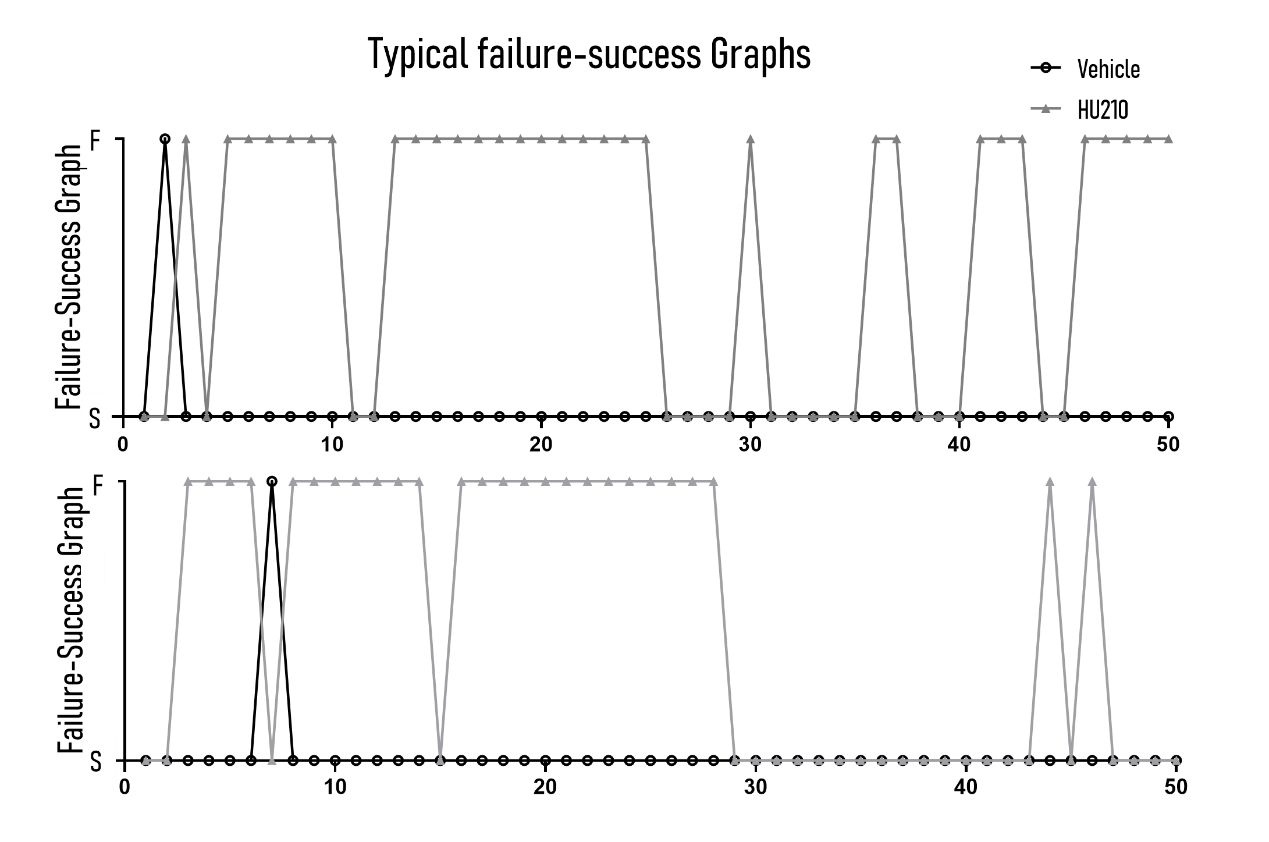
Fig. S6.** **Two** **exemplary learning sessions of mice treated with HU210. Gray and black lines with markers of black circles or triangles depict performance of mice in Vehicle and HU210 groups respectively.** Markers indicate trials sorted into 2 categories according to the outcome. (F (upper) and S (lower) represent failure and success).
