# Supplementary material for: Acute cannabinoids impair association learning via selectively enhancing synaptic transmission in striatonigral neurons
Source: BMC Biol. 2022 May 13;20:108. doi: 10.1186/s12915-022-01307-1 (PMC9102575; doi:10.1186/s12915-022-01307-1)
Supplement: Supplementary file 2 — Additional file 2: Table S1. Oligonucleotide primers and amplicons used in single-cell PCR. Table S2. PCR reaction conditions. [file 12915_2022_1307_MOESM2_ESM.docx]

**Supplemental Tables**

Table 1 Oligonucleotide primers

| Gene | GeneBank accession no. | Primer name | Primer sequence | Product length (bp) |
| --- | --- | --- | --- | --- |
| Gad67 | XM_011239023 | Gad67-F1 | TGTTCCTTTCCTGGTGAGTGC | 296 |
|  |  | Gad67-R1 | GGTAGGAAGCATGCATCTGGT |  |
|  |  | Gad67-F2 | CTTGGCTGTAGCTGACATCTG | 207 |
|  |  | Gad67-R2 | TGCATCAGTCCCTCCTCTCTA |  |
| Drd1 | NM_010076 | Drd1-F1 | TCCGATAGTTGGGCTCATCG | 372 |
|  |  | Drd1-R1 | CTGTTGCAATACCCCCACCC |  |
|  |  | Drd1-F2 | ATAGTTGGGCTCATCGCTGG | 222 |
|  |  | Drd1-R2 | ACCGGGAAGGGGTTCTTCTA |  |
| Drd2 | NM_010077 | Drd2-F1 | AACACACGCTACAGCTCCAA | 325 |
|  |  | Drd2-R1 | TCATGTCCTCAGGGTGGGTA |  |
|  |  | Drd2-F2 | CCCACTGCTCTTTGGACTCA | 152 |
|  |  | Drd2-R2 | GCTTGCGGAGAACGATGTAG |  |

F, forward; R, reverse; 1, primers for first-round PCR; 2, primers for second-round PCR.

Table 2 PCR reaction conditions

| Round | Base mixture | Circles | Temperature | Duration |
| --- | --- | --- | --- | --- |
| 1 | RT product added with 2×PCR Master Mix, ddH2O, and primer 1 (4 μmol/L each) |  | 94 °C | 3 min |
|  |  | 40 | 94 °C | 30 s |
|  |  |  | 59 °C | 1 min |
|  |  |  | 72 °C | 1 min |
|  |  |  | 72 °C | 10 min |
| 2 | An aliquot (2 μL) of the first-round PCR product added with 2×PCR Master Mix, ddH2O, and primer 2 (4 μmol/L each) |  | 94 °C | 3 min |
|  |  | 35 | 94 °C | 30 s |
|  |  |  | 58 °C | 30 s |
|  |  |  | 72 °C | 30 s |
|  |  |  | 72 °C | 10 min |
